# Spatially resolved filament wavefront dynamics

**DOI:** 10.1038/s41598-020-65431-0

**Published:** 2020-06-02

**Authors:** Daniel Thul, Martin Richardson, Shermineh Rostami Fairchild

**Affiliations:** 1 Laser Plasma Laboratory, College of Optics and Photonics, University of Central Florida, 4000 Central Florida Blvd, Orlando, FL 32816 USA; 2 Department of Physics and Space Sciences, Florida Institute of Technology, 3012 Engineering St, Melbourne, FL 32901 USA

**Keywords:** Ultrafast lasers, Nonlinear optics

## Abstract

Spatially resolved wavefront measurements are presented during nonlinear self-collapse and provide the first detailed characterization of wavefront evolution during filament formation. The wavefront dynamics of key nonlinear processes including Kerr self-focusing, ionization and plasma defocusing, and dynamic spatial replenishment are identified and resolved in both the filament core and reservoir regions. These results are analyzed and interpreted with respect to numerical simulations and provide insight into fundamental aspects of filamentation. They also inform applications based on phase manipulation, such as external beam guiding, and present a new method for measuring the nonlinear index of refraction, n_2_.

## Introduction

Filament formation is based on the nonlinear self-collapse of optical beams^1^. Kerr self-focusing (KSF) enables this process by overcoming diffractive spreading when the peak-power of a beam exceeds the critical value for the propagation medium^2,3^. This leads to increasing local intensities that surpass the ionization threshold of the medium^4^. The presence of electrons decreases the local index of refraction, balancing KSF and resulting in an extended, highly confined plasma channel known as a filament^5^. The unique nature of filaments has inspired both practical and academic interest in their capabilities.

The properties of filaments have been well-characterized in several studies through measurements of the plasma channel density, lifetime, length, and diameter^6–10^. The shape^11^, intensity^12^, spectral and temporal modifications^13–15^, and polarization^16^ of the beam formed within the filament have also been studied in detail. The exploration of these properties has produced several novel filament-based applications including white-light seeded parametric amplification^17–19^, pulse compression^20,21^, white-light LIDAR^22,23^, sensing and spectroscopy^24–26^, cloud clearing^27,28^, material modification^29–31^, and guided discharges^32,33^. The input (pre-collapse) wavefront of filament-forming beams has also been exploited to control filament properties after collapse, potentially aiding these applications.

Altering the initial wavefront conditions allows the plasma density^34^, filament length^35^, spectral broadening^36^, and number of filaments^37–40^ to be controlled. It also allows filaments to be organized as arrays, which is useful for nonlinear beam combination and external beam guiding^41–44^. The filament-filament interactions within these arrays are of critical importance and are influenced by the transverse spatial structure of each filament’s plasma, intensity, and wavefront profiles. However, the wavefront progression during collapse and within the filament itself has been largely ignored.

Previous studies of the transverse filament wavefront profile have not measured the full collapse process or have lacked spatial resolution^45–47^. The spatio-temporal nonlinear phase contributions within a filament have been previously been measured interferometrically, but these works do not include measurements of the cumulative wavefront itself^10,48^. This study presents the first spatially resolved filament wavefront measurements throughout the entire filament formation process. These measurements strengthen the understanding of filamentation and are essential to phase-sensitive applications based on filament-filament interactions such as engineered filament arrays and nonlinear beam combining.

## Results and Discussions

Self-collapse of an 800 nm beam is numerically modeled and measured experimentally for pulse energies ranging from 0.6–6 mJ. At 3 mJ, the wavefront dynamics of filament formation are captured and include diffraction, KSF, and plasma defocusing. Varying the pulse energy allows the effects of linear diffraction and KSF to be studied independently. In each case, the initial beam 1/e^2^ diameter is 2.7 mm (1.6 mm FWHM) and the pulse width is 50 fs.

### Simulated filament formation

**Figure 1:**
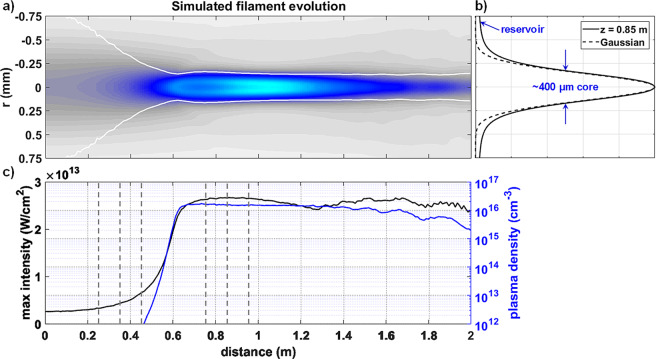
Results of the numerical simulation of a 3 mJ, 50 fs pulse with an initial 1.6 mm FWHM are shown as a function of propagation distance. (**a**) The normalized fluence is plotted on a linear scale with the intensity FWHM indicated by the white curve. (**b**) A radial line profile at z = 0.85 m shows the formation of the Townesian profile. (**c**) The maximum intensity and on-axis plasma density are plotted. The grey lines mark the experimental measurement locations.

The simulated filament formation of a 3 mJ, 50 fs pulse at 800 nm is displayed in Fig. 1. The normalized laser fluence is plotted on a linear scale as a function of radial and axial coordinates in Fig. 1(a) with its FWHM added for reference. A radial line profile of the fluence at z = 0.85 m is provided in Fig. 1(b) and compared with a Gaussian curve to illustrate the formation of the Townesian profile. The corresponding on-axis intensity and plasma density are given in Fig. 1(c). The constant size, intensity, and plasma density observed corresponds to the clamping behavior of filaments that occurs after a balance between KSF and plasma generation has been established^5^.

Wavefront measurements were taken at the six planes indicated by grey dashed lines in Fig. 1(c) and capture the wavefront progression both before and after collapse. The first three measurements are taken before collapse occurs when the numerical plasma density is below 10^12^ cm^−3^ and KSF is the dominant wavefront contribution. Based on the numerical simulation, plasma generation occurs shortly before the measurement plane at 0.75 m. Plasma defocusing is expected to compete with KSF at the three measurement planes located after collapse. The transition from self-collapse to plasma generation and filament formation includes a refocusing cycle that is captured in the measured and simulated wavefront features. This process is described by the accepted theory of dynamic spatial replenishment^49,50^.

During dynamic spatial replenishment the beam intensity rapidly increases during collapse. As the intensity increases, plasma generation occurs and is primarily driven by multi-photon ionization (MPI)^51,52^. The MPI rate is proportional to ${I}^{K}$, where $I$ is the laser intensity and $K=8$ is the number of photons required for ionization in air^5,36^. The ${I}^{8}$ dependence of the ionization rate means that plasma generation occurs suddenly and rapidly, as seen between 0.5 and 0.6 m in the numerical model. This sudden increase in plasma density defocuses the beam and ejects energy from the core. The numerical model captures this dynamic shortly after 0.6 m where the beam diameter increases after reaching a minimum. The energy ejected from the core then slowly returns as KSF and plasma defocusing are balanced and the filament is fully formed. This progression is a fundamental aspect of filament behavior and is captured in the wavefront measurements presented here.

The ${I}^{8}$ dependency of MPI also implies that the transverse profile of the plasma is expected to be smaller and have steeper edges than the Townesian profile generated during filament formation. This aspect of filament formation is also reflected in the following wavefront measurements.

### Wavefront dynamics of competing nonlinearities

**Figure 2:**
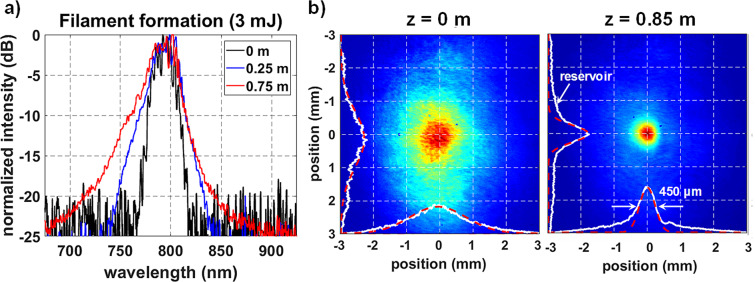
(**a**) The beam’s spectrum was measured as a function of distance for a 3 mJ pulse. The presence of asymmetric spectral broadening was used as a marker of plasma generation. (**b**) The spatial profiles (white lines) are measured before and after collapse and compared to Gaussian curves (red dashed lines). The measurement at 0.85 m shows the formation of the Townesian profile.

The collapse process of the 3 mJ beam was first measured and compared with the numerical model using spectral and spatial profiles. During the initial collapse of the beam, the process of self-phase modulation (SPM) generates symmetric spectral broadening^5,15,36^. Once ionization occurs, the presence of plasma is known to contribute blue-shifted frequencies to the spectral broadening process, leading to asymmetric broadening^5,15^. The symmetrically broadened spectrum observed in Fig. 2(a) at 0.25 m resulted from the SPM process that occurred during self-collapse before the ionization threshold was surpassed. Asymmetric broadening was then observed at 0.75 m indicating the onset of plasma generation. A more thorough discussion of spectral shape is given in Methods. This progression is expected based on the numerical model, which predicts the formation of the plasma channel before the 0.75 m measurement plane. The profiles measured at z = 0 and z = 0.85 m also follow what is expected based on the simulation (Fig. 2(b)). The initially Gaussian profile with a FWHM of 1.6 mm collapses and forms a Townesian profile with a high-intensity core ~400 μm in diameter and a reservoir that extends over 1 mm radially.

**Figure 3:**
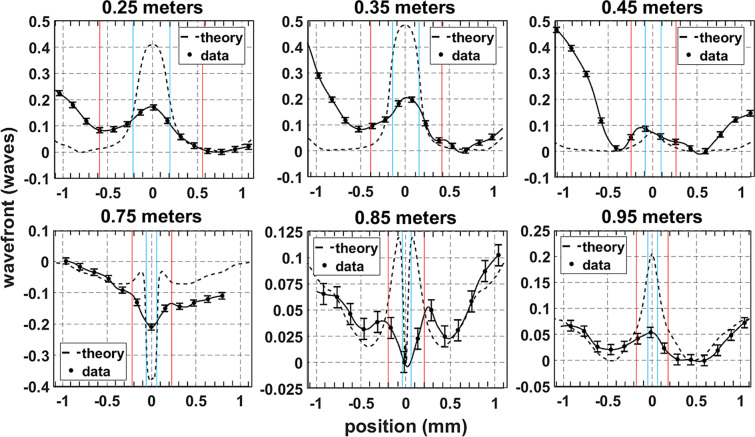
Spatially resolved wavefront measurements are compared to the simulation at several propagation distances for a 3 mJ pulse. The red and blue lines indicate the numerical diameters of the high intensity core (also indicated by white lines in Fig. 1(a)) and the plasma channel, respectively.

The evolution of the transverse wavefront profile calculated in the numerical simulation above is compared to the experimental wavefront profiles in Fig. 3. These measurements were taken using a Shack-Hartmann (SH) wavefront sensor and are shown as a function of transverse spatial coordinate (see Methods). The numerical fluence and plasma diameters are indicated with red and blue lines, respectively, and are used to relate wavefront features to the beam and plasma sizes. In the top row, the measured wavefront progression shows that KSF produced a strong, negative (focusing) curvature in the center of the beam, surrounded by a linearly diffracting background. However, the Kerr wavefront feature was reduced at 0.45 meters, which corresponds to the rapid onset of plasma generation as shown in the numerical model in Fig. 1.

At 0.75 m plasma generation had rapidly increased and introduced a sharp feature in the center of the wavefront with a positive (defocusing) curvature due to plasma defocusing. This measurement coincides with the onset of asymmetric spectral broadening. Note that the plasma feature is ~100 μm in diameter, as is expected based on previous measurements^10,34^. The initial generation of plasma was then combatted by a re-focusing event in the core region. This resulted in a null in the center of the wavefront with peaks on either side of this null, as seen at 0.85 m. This progression follows previous measurements of the nonlinear phase of filaments in silica^48^. The data at 0.95 m shows KSF completely overcoming plasma generation as the re-focusing cycle was completed. The observed balance of KSF and plasma defocusing occurs as the Townesian profile forms, measured at 0.85 m and shown in Fig. 2.

The dual peaks with negative (focusing) curvature measured at 0.85 m indicate that KSF first overcame plasma defocusing at the outside of the core before moving inward. This is expected because the plasma diameter is smaller than the Townesian intensity profile due to the I^8^ dependence of MPI. Also note that the focusing regions extended nearly 0.5 mm radially. This is consistent with dynamic spatial replenishment where energy is initially ejected radially into the reservoir before re-focusing to stabilize the filament.

The wavefront measurement at 0.95 m shows that KSF remained present as the filament fully formed, despite the simulated plasma density remaining constant between 0.75 and 0.95 m. This implies that the measured negative (focusing) curvature in the core was required to sustain the losses due to MPI and plasma absorption. The reservoir directly surrounding the core also had a negative curvature, which served to direct energy into the core. The transition between the core and the reservoir is indicated by the fluence half width, which is ~200 μm at 0.95 m and indicated by the red lines. The reservoir extends radially from 200 μm to 0.5 mm and matches the expected size of the reservoir^5,10,36,47,53–55^. Beyond 0.5 mm the positive wavefront curvature measured indicates that energy outside of the core-reservoir structure was directed away from the filament. This observation is consistent with the balance of nonlinearities required for stable filament formation, which leads to intensity and plasma density clamping. This is an important dynamic of single filaments and has not yet been identified through wavefront measurements.

Despite the spatial resolution of the SH sensor being limited to 150 μm (see Methods), the results show significant qualitative correlation between the simulated and measured wavefront features. The inability to resolve fine wavefront details, specifically the effect of plasma generation, is attributed to this experimental shortcoming. The input beam conditions introduce further disagreement between the measured and simulated data and would persist even with improved spatial resolution. The numerical simulation used here assumes that the input beam has a smooth Gaussian beam profile and an ideal planar wavefront that maintains cylindrical symmetry during propagation. The beam conditions measured at z = 0 show that the Gaussian profile is not perfectly smooth and slightly elliptical (see Methods). The wavefront shape is also only planar to 1/50^th^ of a wave. The wavefront errors near the edge of the beam are most affected by diffraction and are accounted for by the non-ideal input beam conditions. These errors are also present if the following sections, which decouple the primary wavefront effects and isolate the contributions from diffraction and KSF.

### Linear diffraction and propagation of near-critical beams

Linear diffraction plays a key role in the filament formation process and has a large influence on the self-collapse location^1,2^. The effects of diffraction were isolated by reducing the beam energy to 0.6 mJ. At this energy, the pulse propagated near the threshold of self-collapse $\,{P}_{cr} \sim 10\,GW$ (see Methods) with a peak power of $P=\frac{0.6\,mJ}{50fs}=12\,GW$^56^. While this beam had enough peak power to support self-collapse, the expected collapse distance is much greater than the propagation distances measured here. The Marburger equation predicts that a beam with a Rayleigh distance of ${z}_{r}=\pi {w}_{0}^{2}/\lambda$ will collapse at^2^

1 $${L}_{c}=\frac{0.367{z}_{r}}{{\left\{{\left[{\left(\frac{P}{{P}_{cr}}\right)}^{1/2}-0.852\right]}^{2}-0.0219\right\}}^{1/2}}$$

The 12 GW input beam with a waist diameter of $2{w}_{0}=2.7{\rm{mm}}$ had an expected collapse distance of ${L}_{c}=13.6{\rm{m}}$, well beyond the maximum measurement distance of 1.15 m. Therefore, linear diffraction was expected to have the strongest influence on the wavefront shape with minimal contribution from KSF. The theoretical wavefront shape based on linear diffraction alone is given by the well-known formula^57^

2 $${W}_{e}^{L}(r,z)=\frac{1}{2\pi }\cdot \frac{{k}_{0}{r}^{2}}{2R(z)}=\frac{{r}^{2}}{2\lambda \left(z+\frac{{z}_{r}^{2}}{z}\right)}$$

The estimated linear wavefront contribution ${{\rm{W}}}_{{\rm{e}}}^{L}$ is given in waves and is a function of the radial coordinate $r$ for a given propagation distance ${\rm{z}}$.

**Figure 4:**
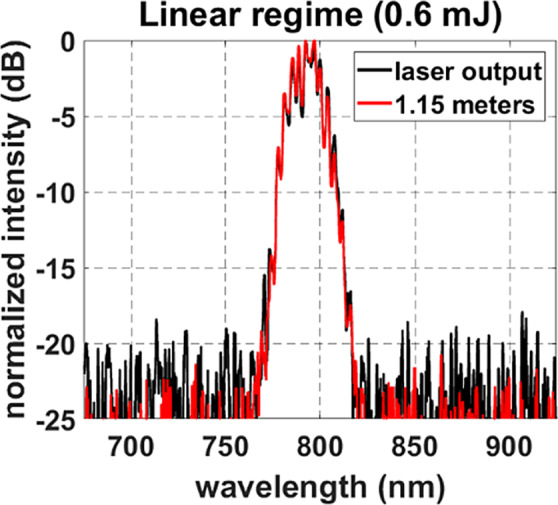
Spectral measurements taken at the laser output (black line) and after 1.15 m of propagation for the 0.6 mJ beam (red line). The spectra are nearly identical and do now show broadening.

The dominant role of linear diffraction was confirmed by the lack of spectral broadening observed along propagation due to the minimal presence of nonlinearities. The output spectrum of the laser is compared to the spectrum of the 0.6 mJ pulse at the final propagation distance of 1.15 m in Fig. 4 and shows a lack of broadening.

**Figure 5:**
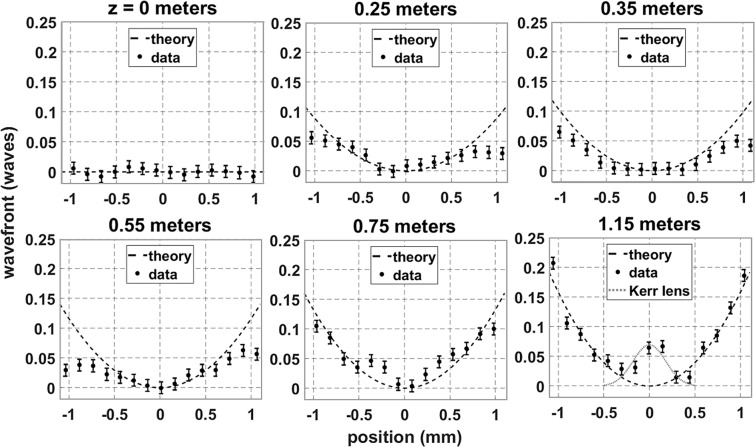
The input wavefront shows that the beam is initially collimated within the resolution of the SH sensor. The wavefront progression at 0.6 mJ followed the shape estimated using Eq. 2, confirming that linear diffraction dominated the wavefront dynamics in this regime. The Kerr effect only began to influence the wavefront at the furthest propagation distance.

The expected and measured wavefronts are compared as a function of distance for the 0.6 mJ pulse in Fig. 5. The first measurement at z = 0 m shows the optimized planar wavefront achieved at the laser output. Note that the wavefront deviation was below 1/50^th^ of a wave, which was the resolution limit of the measurement (see Methods). As the beam propagated, the measured wavefront evolution followed the quadratic contribution predicted by Eq. (2).

**Figure 6:**
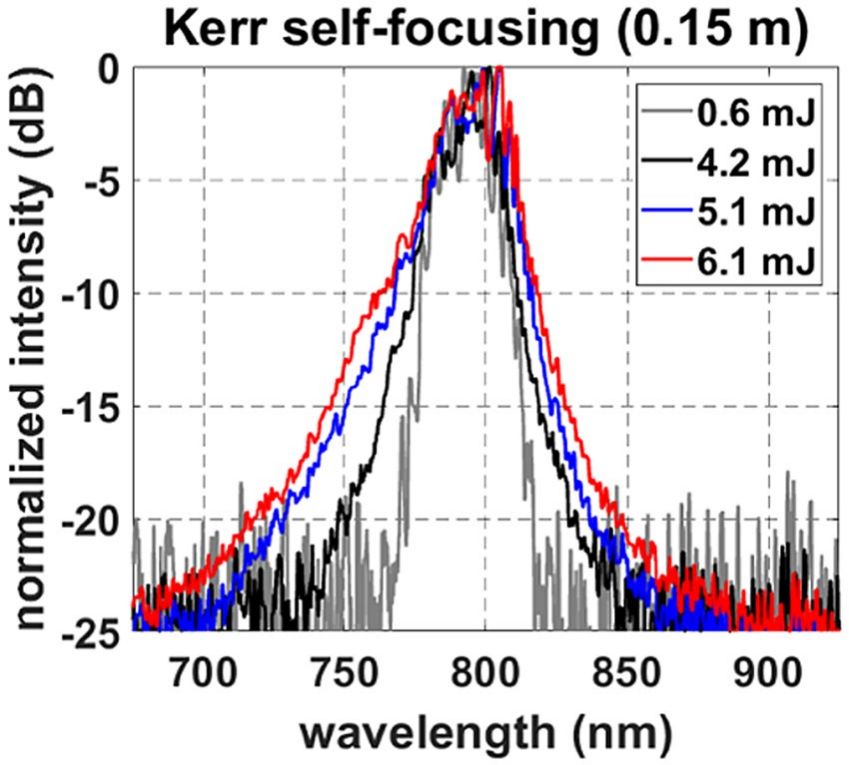
Spectral broadening data taken at 0.15 m. Broadening increased symmetrically as the energy increased, indicating the presence of SPM that occurred during KSF at the beginning of collapse.

**Figure 7:**
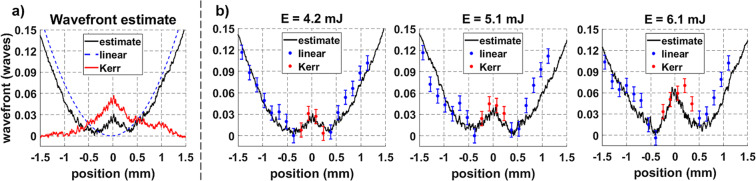
(**a**) The estimated wavefront shape is found by combining the linear diffraction term (dashed blue line) and the Kerr term (solid red line), as shown in Eq. (4). The calculated and measured wavefronts at 0.15 m are compared as a function of energy in (**b**). These measurements show the effect of diffraction (blue) and KSF (red), which are used to calculate n_2_.

**Figure 8:**
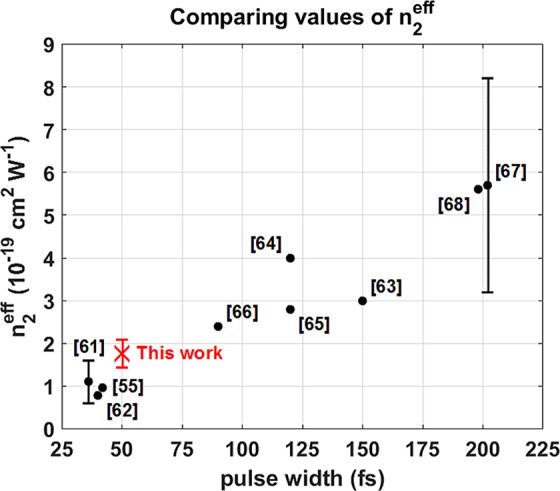
The distribution of n_2_ measurements aligns with what is found in the literature and is consistent with what is expected for a 50 fs pulse.

Equation (2) assumes that the wavefront at z = 0 is perfectly flat, which was not the case for the measured beams based on the wavefront sensitivity of the SH sensor (see Methods). As previously stated, this limitation of the measurement technique is likely the source of the disagreement between theoretical and measured data observed near the edges of the beams at 0.25, 0.35, and 0.55 m. Also note that the wavefront measured at 1.15 m began to show some evidence of self-focusing in the center of the beam, highlighted by the Gaussian fit shown in red (the Kerr effect is proportional to intensity and is expected to be Gaussian based on the input beam profile^1,2,5^). This is not unexpected since the peak power is slightly above the collapse threshold of 10 GW and Eq. (1) predicts a collapse location of 13.6 m. The measurement at 1.15 m captured the beginning of this collapse process, but the strength of KSF is minimal based on the lack of spectral broadening. The following section investigates the transition from linear diffraction to KSF as the pulse energy is increased.

### Resolving the Kerr effect and measuring n_2_

The driving force behind self-collapse is the Kerr effect, which overcomes diffraction as the dominant wavefront contribution for beams above the critical power. This is observed at 1.15 m for the near-critical beam as KSF slowly accumulates. At the higher energies used here, the transition to self-focusing will occur much earlier and is observed at the first measurement plane of 0.15 m. The effect of KSF is cumulative with distance and can be estimated using the following integral^5^:

3 $${W}_{e}^{K}(r,z)=\frac{1}{\lambda }{\int }_{0}^{z}{n}_{2}I(r,z\text{'})dz\text{'} \sim {n}_{2}I(r,z)\cdot \left(\frac{z}{\lambda }\right)$$

The approximation of the integral in Eq. (3) is possible due to the short propagation distance; the intensity profile $I(r,z)$ is assumed to have retained its shape within the first 0.15 m of propagation. The measured intensity profile, pulse energy, and pulse width were used to calculate $I(r,z)$. The estimated Kerr component was added to the linear component given in Eq. (2) to yield the total wavefront estimate

4 $${W}_{e}(r,z)={W}_{e}^{L}+{W}_{e}^{K}=\left(\frac{{r}^{2}}{2({z}^{2}+{z}_{r}^{2})}+{n}_{2}I(r,z)\right)\cdot \left(\frac{z}{\lambda }\right)$$

The wavefront estimate ${W}_{e}(r,z)$ is particularly useful because it can be found using measured quantities. The value of n_2_ is estimated by comparing ${W}_{e}(r,z)$ to measured wavefront profiles at three different energies.

The following measurements were taken at 0.15 m, before the beam intensity surpasses the ionization threshold of air. A key indicator of KSF is symmetric spectral broadening produced by SPM under nonlinear focusing conditions^5,15,36^. Plasma generation also causes spectral broadening, but its contribution is not symmetric and mostly blue-shifted^5^. Figure 6 shows the spectra measured at 0.15 m. The symmetric increase in spectral width indicates that these measurements captured the self-collapse stage of filament formation before plasma generation occurred. Evaluation of the spectral shape is further discussed in Methods.

The lack of plasma at 0.15 m is also predicted by estimating the peak laser intensity generated by the most energetic pulse at this location. This is done using the pulse energy, 1/e^2^ spot size, and pulse duration as

5 $$I=\frac{{E}_{p}}{\pi w{(z=0.15m)}^{2}\tau } \sim 7\cdot {10}^{12}W\cdot c{m}^{-2}\ll {I}_{th},$$

where the ionization threshold ${I}_{th} \sim (1-3)\cdot {10}^{14}\,W\cdot c{m}^{-2}$ for femtosecond pulses at 800 nm in air^58–61^. The laser intensity under these conditions is roughly 2 orders of magnitude below the ionization threshold and any effects of plasma can be neglected.

The wavefront shape measured at 0.15 m for each energy condition is compared to the corresponding estimate found using Eq. (4). An example of this estimate is plotted in Fig. 7 and shows that there is a strong resemblance between the measured and estimated wavefront profiles. As in the previous section, linear diffraction was the primary wavefront contribution near the edge of the beam (shown in blue). The Kerr effect produced an additional feature in the center of the beam where the intensity is largest as the filament core eventually forms (shown in red). As expected, this feature has a negative (focusing) curvature and is more prominent at higher energies.

Comparing the measured and estimated wavefront shapes allows the value of n_2_ in air to be estimated. This is done by equating the measured ($${W}_{m})$$ and estimated ($${W}_{e}$$) wavefronts and re-arranging Eq. (4) as

6 $${n}_{2}=\frac{1}{n}\cdot \frac{\lambda }{z}\mathop{\sum }\limits_{i}^{n}{\left(\frac{{W}_{m}-{W}_{e}^{L}}{I}\right)}_{i}$$

The measurements used for this calculation are shown in red in Fig. 7 and lie near the center of the beam where the Kerr effect was the most pronounced. The value of n_2_ calculated in Eq. (6) is compared to previous measurements in Fig. 8^56,62–69^.

The measurements yielded an average value of ${n}_{2}=1.75\pm 0.33\cdot {10}^{-19}c{m}^{2}{W}^{-1}$, which is consistent with the trend observed in previous measurements. In general, the effective nonlinear refractive index in air depends on contributions from both the instantaneous and delayed Kerr effect. The increase in effective n_2_ values observed in Fig. 8 is attributed to an increase in non-instantaneous Kerr contributions at longer pulse widths^5,56,63^.

## Conclusion

This work presents the first spatially resolved wavefront measurements that span the entire self-collapse process of filaments while simultaneously capturing the core and reservoir wavefront dynamics. The numerical model used is representative of the measured data and allows the wavefront contributions of linear diffraction, KSF, and plasma defocusing to be identified. The measurements show that the wavefront plays a critical role in the formation of the core-reservoir structure and follows the theory of dynamic spatial replenishment. These results also utilize the established theories of linear diffraction and KSF to perform direct measurements of n_2_ and characterize linear diffraction near the edge of the filament. This work advances the fundamental understanding of filament formation and provides a new diagnostic tool to be used in future phase-sensitive filament applications.

## Methods

### Characterizing filament wavefronts

**Figure 9:**
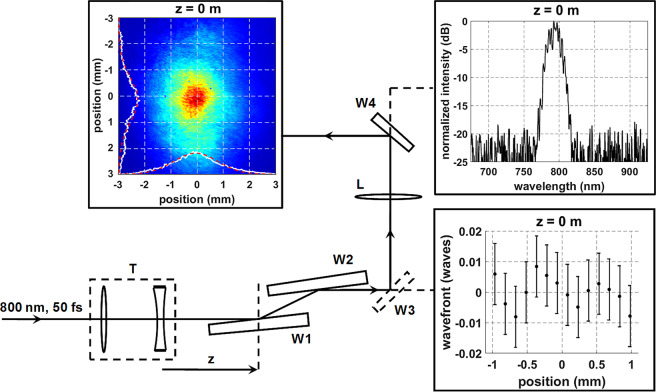
Experimental setup. The laser output was resized from ~15 mm to 2.7 mm using a telescope T and formed a filament under self-collapse conditions. The beam was characterized at several locations z. The measurement plane is indicated with a dashed line. The SH sensor measured the wavefront directly after nonlinear propagation was arrested using wedges W1 and W2. A removable wedge W3 allowed the intensity profile to be measured after one-to-one imaging using a 300 mm lens L. A final wedge W4 was used to further attenuate the beam before imaging the beam profile on the CCD. The light transmitted through W4 was used for spectral measurements.

The following experiments were performed using the Multi-Terawatt Femtosecond Laser, a Ti:sapphire source producing 50 fs pulses centered at 800 nm^70^. Pulse energies ranging from 0.6–6 mJ were studied during propagation under self-collapse conditions. A commercial Shack-Hartmann sensor (ThorLabs model: WFS150-7AR)^71^ was used to spatially resolve the beam’s transverse wavefront structure as a function of propagation distance. The wavefront profile is reconstructed by stitching the local wavefront tilt sampled by each sub-aperture of the micro-lens array in the SH sensor. The resultant wavefront deflection (the phase difference between the measured wavefront and a planar wavefront at the propagation distance z) is given in waves relative to the 800 nm laser wavelength and is time-averaged over the entire temporal pulse width. Spatial intensity and spectral measurements were also captured to contextualize the measured wavefronts within the filament formation process. The filaments were characterized using the setup shown in Fig. 9.

The initial beam size and wavefront flatness are set by the telescope. Re-collimating the beam to a 1/e^2^ diameter of 2.7 mm had multiple advantages. First, it allowed the beam to fit on the SH sensor. This is ideal because the beam could be projected directly on the sensor, eliminating wavefront distortion due to the passage through any imaging optics^72^. The telescope also allowed optimization of the initial wavefront by ensuring that it was as planar as possible. This optimization was limited by the sensitivity of the SH sensor, which is $\lambda /50$ after averaging 10 or more measurements^71^. In this study 100 wavefront measurements were averaged at each measurement condition. The initial wavefront was optimized directly after the laser output by adjusting the spacing between the lenses in the de-magnifying telescope. In addition, the use of a smaller beam allowed the filaments to be studied under self-collapse conditions at accessible distances. Larger beams form filaments at much longer distances, without the use of any focusing optics, which is not practical for table-top experiments^5^. Studying the wavefront in the self-collapse regime was particularly valuable since the wavefront shape was influenced exclusively by the filament formation process, without the contribution of a focusing lens^47^.

After re-collimation, the pulse propagated a distance z to the measurement plane (dashed line). Two wedges at grazing angles were placed at the measurement plane in order to attenuate the beam and arrest nonlinear propagation, avoiding the use of neutral density filters and minimizing wavefront deformations. The SH was placed as close as possible (50 mm) to the measurement plane (W3 removed). This extra propagation distance was corrected for by adding 50 mm to the z terms in Eqs. (2) and (4) that account for linear diffraction. With W3 in place, the spatial profile was imaged 1:1 on the CCD (Imaging Source model: DMK 22BUC03) using a 300 mm lens (L). Spectral measurements were also taken using the residual light going through a wedge (W4). The wedges were monitored for damage after each measurement (100 shots at 10 Hz). If damage occurred, the wedges were shifted and the measurement was repeated. However, this issue was mostly avoided due to the large wedge width of six inches.

The ability to accurately capture wavefront features was determined by the spatial resolution and the wavefront sensitivity of the SH sensor. The 150 μm pitch of the microlens array used to spatially sample the wavefront limits the minimum resolvable wavefront feature. In addition, the wavefront sensitivity prevents the formation of an ideal planar wavefront at the telescope output. These effects limit the accuracy and resolution of the presented measurements. Finally, the input beam in not perfectly cylindrically symmetric as assumed in the numerical simulation. The minor ellipticity is a potential source of disagreement between measured and simulated data.

### Spectral broadening

Evaluations of spectral broadening were key to determining the stage of filament formation during this study. Claims of symmetric spectral broadening are given relative to the original spectrum. Broadening through self-phase modulation will not be perfectly symmetric unless the input spectrum is also perfectly symmetric^73^, which is not the case here. Instead, our assessments depend upon proportional widening of the spectrum on both sides as energy is increased. As such, the broadening observed in Fig. 2(a) between 0.25 and 0.75 m is labeled as asymmetric because it almost exclusively occurs on the ‘blue’ side of the spectrum. In contrast, both the ‘blue’ and ‘red’ sides widen proportionally in Fig. 6 during symmetric spectral broadening. This assessment has been utilized in previous publications^5,14,15,36^ and is suitable in this context.

### Numerical model

Filaments require a dynamic balance between diffraction, KSF, and plasma defocusing to sustain propagation. However, there are a host of additional processes that must be considered to fully understand filament formation. For this reason, a numerical simulation is required to properly model the complex nature of filaments. Filament formation was simulated by solving the nonlinear Schrödinger equation using a 2D + 1 split-step Fourier method, given in normalized coordinates as

7 $$\begin{array}{rcl}\frac{\partial \varepsilon }{\partial z} & = & \frac{i}{2k}{\nabla }_{\perp }^{2}\varepsilon -i\frac{k{\prime\prime} }{2}\frac{{\partial }^{2}\varepsilon }{\partial {t}^{2}}+i{k}_{0}{n}_{2}(1-f){|\varepsilon |}^{2}\varepsilon \\  &  & +i{k}_{0}{n}_{2}ft[{\int }_{-\infty }^{t}R(t-t{\prime} ){|\varepsilon (t{\prime} )|}^{2}dt{\prime} ]\varepsilon \\  &  & -\frac{\sigma }{2}(1+i{\omega }_{0}{\tau }_{c})\rho \varepsilon -\frac{{\beta }_{K}}{2}{|\varepsilon |}^{2K-2}\left(1-\frac{\rho }{{\rho }_{nt}}\right)\varepsilon \end{array}$$

The first two terms on the right-hand-side describe linear diffraction and dispersion while the following four terms account for the instantaneous Kerr nonlinearity, the rotational Raman Kerr contribution, plasma absorption and refraction, and multi-photon ionization. The radial distance from the propagation axis and the local time of the pulse are resolved at each propagation distance with assumed rotational symmetry in the transverse plane. The nonlinear terms are computed in the spatio-temporal domain while linear diffraction and dispersion are calculated in their respective Fourier domains. A more thorough description of the numerical model used can be found in related publications^15,36^. The simulation results were obtained using a 3 mJ, 50 fs pulse at 800 nm with a 1.6 mm full-width at half maximum (FWHM), which corresponds to a 2.72 mm 1/e^2^ diameter.

The choice for n_2_ in the simulation was informed by previous measurements of the critical power using ~50 fs pulses, which is defined as

8 $${P}_{cr}=\alpha \frac{{\lambda }^{2}}{8\pi {n}_{0}{n}_{2}}$$

and depends on the wavelength $\lambda$, linear and nonlinear refractive indices ${n}_{0}$ and ${n}_{2}$, and a factor $\alpha$ defined by the initial beam profile ($\alpha =3.79$ for a Gaussian beam)^1–3^. The importance of accounting for pulse width is highlighted in the comparison of measured effective n_2_ values shown in Fig. 8. Previous studies using pulse widths between 36 and 42 fs have measured critical powers ranging from 6–16 GW, which correspond to an effective nonlinear refractive index between 0.6∙10^−19^ and 1.6∙10^−19^ cm^2^ W^−1^ based on Eq. 8^56,62,63^. The average n_2_ found in these three studies is ~10^−19^ cm^2^ W^−1^ and is used in the simulations presented here. This value of n_2_ is also used in Eq. (8) to calculate the value of ${P}_{cr} \sim 10\,GW$ used in Eq. 1.

## Data Availability

The datasets generated and analyzed during the current study are available from the corresponding author on reasonable request.
